# Extracellular Vesicles as Communicators of Senescence in Musculoskeletal Aging

**DOI:** 10.1002/jbm4.10686

**Published:** 2022-10-13

**Authors:** Monica Correa Alfonzo, Ahmed Al Saedi, Sadanand Fulzele, Mark W. Hamrick

**Affiliations:** ^1^ Universidad Central del Caribe Bayamón Puerto Rico USA; ^2^ Department of Pediatrics, Harvard Medical School and Division of Endocrinology Boston Children's Hospital Boston Massachusetts USA; ^3^ Medical College of Georgia Augusta University Augusta Georgia USA

**Keywords:** EXOSOMES, MALAT1, miR‐34a, OSTEOARTHRITIS, OSTEOPOROSIS, SARCOPENIA

## Abstract

Extracellular vesicles (EVs), including exosomes and microvesicles, are released by numerous cell types. EVs are now acknowledged as playing a critical role in cell–cell communication in healthy aging as well as in age‐related diseases. Recently it was shown that senescence, a key hallmark of aging, increases the secretion of EVs. Moreover, EVs can transport proteins and microRNAs (miRNAs) that are key components of the senescence‐associated secretory phenotype (SASP). Here we review evidence that SASP‐related miRNAs are involved in musculoskeletal degeneration with aging. Specifically, senescence‐related miRNAs are elevated in EVs released by skeletal muscle myocytes and fibro‐adipogenic progenitor cells with aging and disuse atrophy, respectively. Many of these same senescence‐related miRNAs are detected in EVs from the synovial fluid of patients with osteoarthritis, and these miRNAs can contribute to cartilage degeneration. Finally, senescence‐associated miRNAs are secreted from bone marrow–derived stem (stromal) cells impacting neighboring hematopoietic stem cells and circulating in the blood. The senescence‐associated miRNA mir‐34a, which is known to target Wnt and Notch pathways as well as the cell survival factors Sirt1 and Bcl2, is detected in EVs from human and animal subjects with muscle atrophy, bone loss, and osteoarthritis. These findings suggest that suppressing the secretion of EV‐derived, senescence‐related miRNAs, such as miR‐34a, or increasing levels of competing endogenous long noncoding RNAs, such as MALAT1 that inhibit miR‐34a, may help to improve musculoskeletal function with aging. © 2022 The Authors. *JBMR Plus* published by Wiley Periodicals LLC on behalf of American Society for Bone and Mineral Research.

## Graphical abstract

Senescent cells secrete more extracellular vesicles (EVs) than healthy cells. EVs released by senescent muscle, bone, and cartilage cells carry senescence‐associated microRNAs that can induce senescence in neighboring cells, driving tissue degeneration.

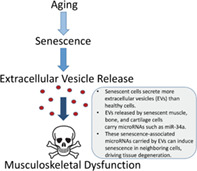

## Introduction

Degenerative musculoskeletal diseases, such as osteoporosis, osteoarthritis, and sarcopenia, contribute to significant morbidity and disability in populations around the world. The prevalence of these diseases increases with age, and as the size of the older population increases globally, these diseases place a greater burden on patients, their families, and public health systems.^(^ ^1^ ^)^ It is now understood that multiple age‐related disorders affecting various organs and tissues may have some common underlying causes. Specifically, increased levels of oxidative stress and circulating inflammatory cytokines can negatively impact multiple organs and tissues simultaneously. This fact is underscored by parabiosis studies demonstrating that, on the one hand, circulating factors in aged animals can suppress regenerative processes in young animals and that, on the other hand, young blood can enhance regeneration and cognition in aged animals.^(^ ^2^, ^3^, ^4^ ^)^ Numerous circulating factors have been implicated in these systemic changes, such as transforming growth factor beta 1, Notch family members, and age‐associated factors like Klotho.^(^ ^2^, ^5^ ^)^

Recently it has been suggested that the cargo of circulating extracellular vesicles (EVs), including exosomes and microvesicles, is significantly altered with age and with age‐related diseases, such as Alzheimer's disease, and that a more “youthful” EV cargo could reverse some degenerative, age‐related changes.^(^ ^6^, ^7^ ^)^ A key factor driving EV release with aging is senescence. Senescent cells, which have exited the cell cycle and express markers such as p16 and p21, accumulate with age. These cells can promote senescence in neighboring cells through the secretion of factors collectively termed the senescence‐associated secretory phenotype (SASP).^(^ ^8^ ^)^ Notably, a large number of SASP proteins are detected in EVs, and miRNAs associated with senescence are also abundant within EVs (Table 1).^(^ ^8^, ^9^, ^10^, ^11^, ^12^ ^)^ Furthermore, senescence itself is found to induce EV secretion.^(^ ^13^, ^14^ ^)^ In fact, senescent cells secrete 20% to 30% more EVs than nonsenescent cells.^(^ ^15^ ^)^ These findings suggest that EVs may be important communicators of senescence in both a local and systemic manner.

**Table 1:** Proteins Previously Identified in Secretory Profile of Senescent Cells^(^ ^8^ ^)^ and microRNAs Previously Associated with Cellular Senescence^(^ ^9^, ^10^, ^11^ ^)^ Also Identified in EVs (Vesiclepedia; ref. ^(^ ^12^ ^)^)

SASP proteins identified in EVs	Senescence‐associated microRNAs identified in EVs
IL‐6	let‐7 family
IL‐7	miR‐107
IL‐13	miR‐141
IL‐15	miR‐146a
IFN‐γ	miR‐181a
VEGF	miR‐204
SDF‐1	miR‐21
IGFBP‐2, −3, −4, −6, −7	miR‐210
MMP‐1, −3, −10, −13, −14	miR‐26b
TIMP‐1	miR‐34a
TIMP‐2	miR‐335
Cathepsin B	miR‐424
ICAM‐1	
ICAM‐3	

We previously showed that skeletal muscle cells secrete senescence‐associated miRNAs in EVs with age^(^ ^16^ ^)^ and disuse atrophy,^(^ ^17^ ^)^ and others have identified similar changes in EVs derived from senescent chondrocytes.^(^ ^18^ ^)^ Here we review the evidence for EVs as communicators of senescence in musculoskeletal tissues and identify potential interventions to target these EVs as an approach for the prevention and treatment of age‐related musculoskeletal diseases. Original research papers were found using National Institutes of Health (NIH) PubMed (accessed on June–July 2022), including search terms such as “senescence + osteoarthritis + extracellular vesicles,” “senescence + osteoporosis + extracellular vesicles,” and “senescence + sarcopenia + extracellular vesicles.” Other searches also included “miRNA” as an additional search term. Papers were selected based on relevance and a preliminary abstract screening. A number of the articles were already present in the authors' libraries and were included where appropriate.

### EVs, senescence, and muscle atrophy

Myoblasts and mature myotubes are known to secrete EVs both in vitro and in vivo.^(^ ^19^ ^)^ EV secretion is increased with exercise,^(^ ^20^, ^21^ ^)^ possibly through membrane (sarcolemma) damage that leads to calcium influx and exosome release.^(^ ^22^ ^)^ Exposure of cells to inflammatory cytokines with aging is associated with accumulation of the sphingolipid ceramide and ceramide can, in turn, induce senescence.^(^ ^23^, ^24^ ^)^ Importantly, ceramide accumulation also triggers exosome secretion,^(^ ^25^ ^)^ and exosomes are known to be highly enriched in ceramide.^(^ ^26^ ^)^ Muscle‐derived EVs are therefore likely to be secreted in conditions associated with muscle contraction and muscle aging.

**Fig. 1:**
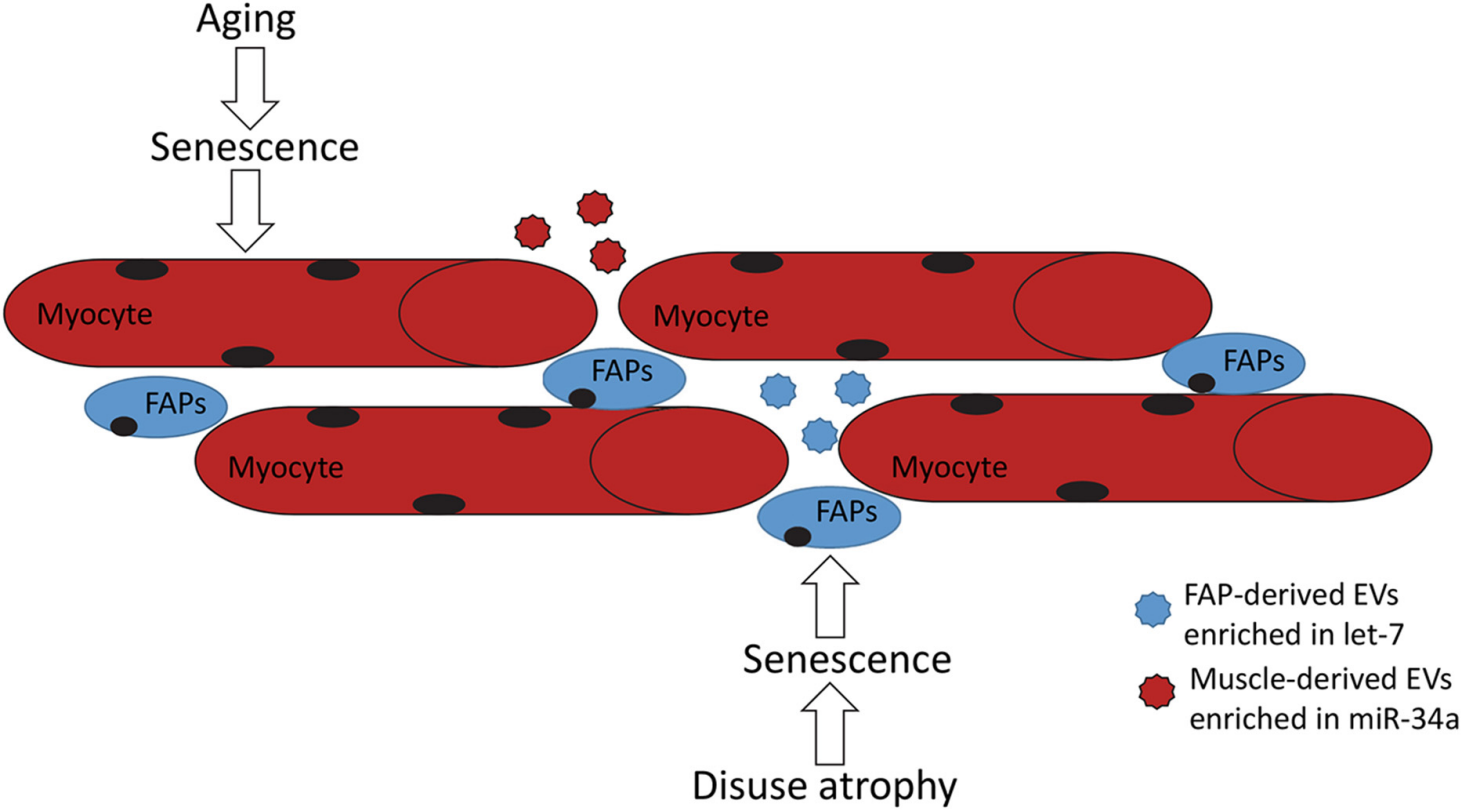
Aging and disuse atrophy induce senescence in skeletal muscle and fibro‐adipogenic progenitor cells (FAPs). Age‐assocated senescence stimulates EV release, and these EVs transport senescence‐associated microRNAs, such as miR‐34a (red). Disuse atrophy can also induce senescence in FAPs, which release EVs carrying senescence‐associated microRNAs such as let‐7 family members (blue). These EVs can communicate a senescent phenotype to neighboring cells and also enter the circulation.

We have found that the senescence‐associated miRNA miR‐34a accumulates with age in mouse skeletal muscle,^(^ ^16^ ^)^ and others have observed the same age‐related increase in miR‐34a in human skeletal muscle.^(^ ^27^ ^)^ We previously used alpha sarcoglycan (SGCA) as a surface marker to isolate muscle‐derived EVs from serum and found that circulating, muscle‐derived EVs from older animals are enriched in miR‐34a.^(^ ^16^ ^)^ Mir‐34a is associated with aging and senescence in other tissues, including the heart^(^ ^28^ ^)^ and brain,^(^ ^29^ ^)^ and is known to target Wnt and Notch pathways, as well as cell survival factors such as Sirt1 and Bcl2. Interestingly, miR‐34a overexpression in muscle cells leads to ceramide accumulation,^(^ ^30^ ^)^ and we have found that miR‐34a overexpression in muscle cells leads to their release via EVs.^(^ ^16^ ^)^ Together these findings suggest that factors such as oxidative stress that induce miR‐34a expression may in turn trigger the export of miR‐34a via EVs by stimulating ceramide accumulation (Fig. 1). Local and circulating EV‐derived miR‐34a may then communicate senescence to recipient tissues such as bone.^(^ ^16^ ^)^ These findings suggest that muscle‐derived EVs may contribute to age‐related bone loss via a bystander effect, whereby senescent cells induce senescence in nearby cells and tissues.

Myoblasts and myocytes are not the only cells in skeletal muscle that secrete EVs. Fibro‐adipogenic progenitor cells (FAPs) are known to have a substantial effect on muscle satellite cells via their secretome.^(^ ^31^ ^)^ Recently it was shown that small molecule therapy using histone deacetylase inhibitors could “tune” FAPs to release EVs that promote muscle regeneration via their miRNA cargo.^(^ ^32^ ^)^ We utilized a single‐hindlimb immobilization model to demonstrate that muscle disuse atrophy could induce senescence in FAPs. Specifically, 2 weeks of disuse increases the expression of interleukin‐1 beta (IL‐1β), a key SASP factor,^(^ ^8^ ^)^ as well as its receptor IL‐1R.^(^ ^33^ ^)^ IL‐1β colocalizes with the senescence marker p16 in FAPs in this immobilization model.^(^ ^33^ ^)^ We have used PDGFRα as a surface marker to isolate FAP‐derived EVs from whole skeletal muscle.^(^ ^17^ ^)^ MiRNA profiling of these FAP‐derived EVs reveals that a number of senescence‐associated miRNAs, including let‐7 family members and miR‐181a, are increased in muscle with disuse immobilization^(^ ^17^ ^)^ (Fig. 1). These miRNAs target mitochondrial membrane solute carriers, such as SLC25A3, which decreases in muscle with immobilization^(^ ^17^ ^)^ and in muscle biopsies from sarcopenic patients with hip fracture.^(^ ^34^ ^)^ Future studies might be directed at identifying EVs positive for either SGCA or PDGFRα in circulation and in bone marrow interstitial fluid to determine their role in age‐related tissue dysfunction.

### Osteoarthritis and senescence‐associated EVs

**Fig. 2:**
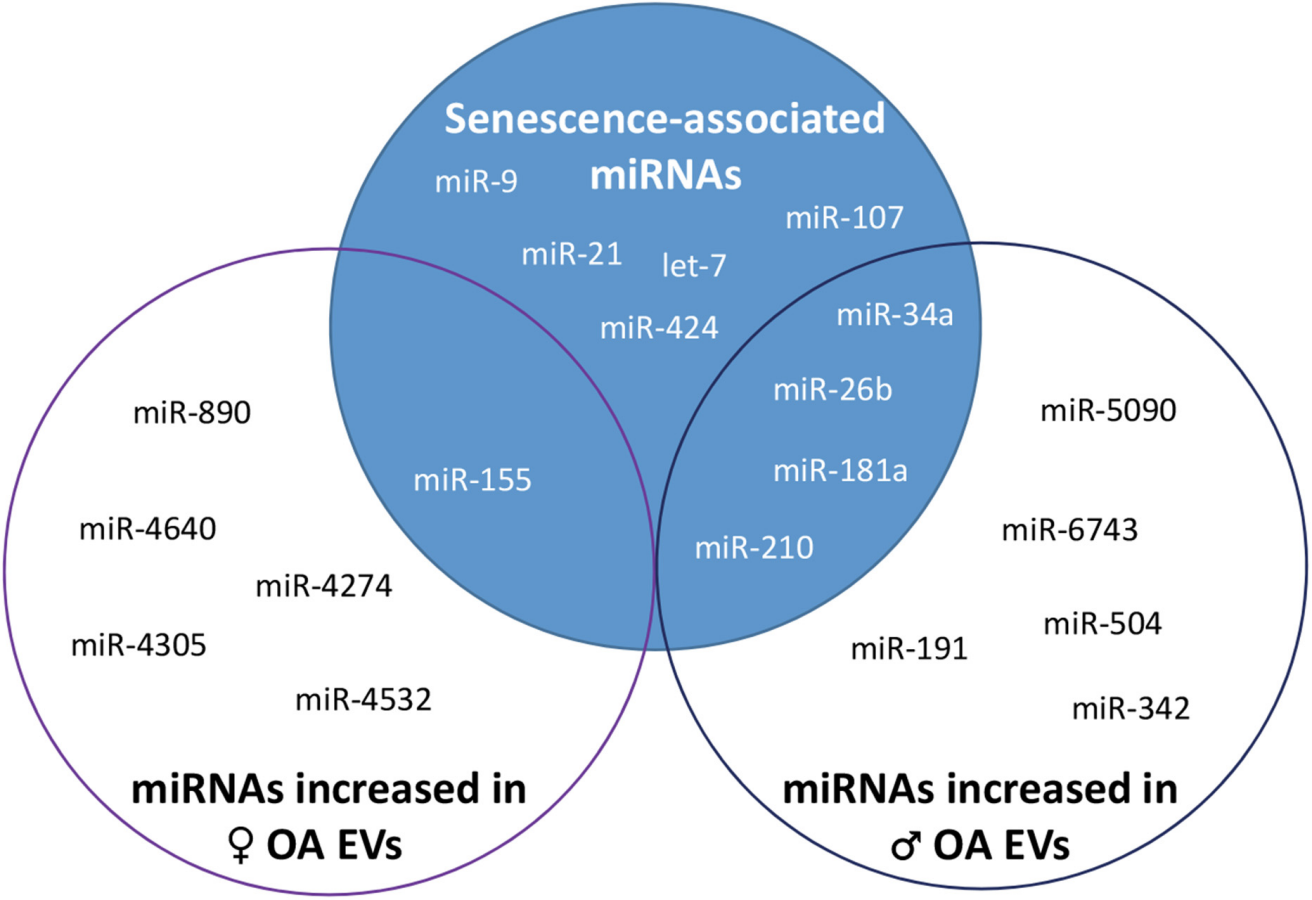
Venn diagram showing senescence‐associated microRNAs detected in EVs from the synovial fluid of male and female osteoarthritis (OA) patients.^(^ ^37^ ^)^ Males tend to express more senescence‐associated miRNAs than females, indicating that the cellular changes that occur between males and females with aging and joint degeneration are likely to differ.

EVs are known to circulate in synovial fluid and are released by chondrocytes and synoviocytes.^(^ ^35^, ^36^ ^)^ We^(^ ^37^ ^)^ and others^(^ ^38^ ^)^ have found that a number of senescence‐associated miRNAs, such as miR‐34a, mirR‐155, and miR‐181a, are identified in synovial fluid from patients with OA (Fig. 2). Importantly, there are noticeable sex differences in the microRNA profile of EVs derived from the synovial fluid of OA patients (Fig. 2), with males expressing more typical senescence‐associated miRNAs than females.^(^ ^37^ ^)^ Our work revealed elevated levels of miR‐34a in samples from male OA patients, whereas other studies found increased miR‐34a in plasma, cartilage, and synovium of both male and female OA patients.^(^ ^39^ ^)^ Importantly, intra‐articular injection of miR‐34a alone can induce an OA phenotype in rodents,^(^ ^39^ ^)^ consistent with other studies showing that EV‐derived miR‐34a can communicate senescence in chondrocytes, whereas senolytic therapy attenuates this effect.^(^ ^18^ ^)^ These findings suggest that EVs are key communicators of senescence in the limb joint microenvironment and may contribute to the development and progression of degenerative joint disease.^(^ ^40^ ^)^ This conclusion is further supported by data showing that EVs enriched in Connexin 43 released from senescent chondrocytes “spread” senescence to neighboring cells.^(^ ^41^ ^)^

### EVs as communicators of senescence in aging bone microenvironment

The balance of bone formation and bone resorption is mediated by crosstalk among a variety of cell types in the bone marrow microenvironment, including bone marrow stromal (stem) cells (BMSCs), osteocytes, osteoblasts, osteoclasts, hematopoietic stem cells, and bone marrow adipocytes. These various cell types have all been found to secrete EVs that can impact neighboring cells.^(^ ^42^, ^43^ ^)^ For example, the muscle‐derived factor myostatin can stimulate the release of osteocyte‐derived EVs carrying the miRNA miR‐218, which in turn suppresses bone formation by osteoblasts.^(^ ^44^ ^)^ Increases in senescence‐associated miRNAs with age in EVs are associated with a loss of bone marrow stem cells and impaired bone formation.^(^ ^45^ ^)^ BMSCs are known to secrete EVs enriched in miR‐31, which can stimulate bone resorption by osteoclasts,^(^ ^46^ ^)^ whereas osteoclast‐derived EVs carrying miR‐214 can suppress bone formation by osteoblasts.^(^ ^47^ ^)^ Several studies have found miR‐34a to increase with age in EVs secreted by BMSCs,^(^ ^48^, ^49^ ^)^ and miR‐34a suppresses osteoblast differentiation as well as bone formation by differentiated osteoblasts.^(^ ^50^, ^51^ ^)^ Total circulating levels of miR‐34a (not solely EV‐derived miR‐34a) are inversely correlated with bone mineral density in older adults,^(^ ^52^ ^)^ and circulating miR‐34a is increased with ovariectomy and positively correlated with bone loss in rodents.^(^ ^53^ ^)^ Furthermore, EVs from aged bone matrix transfer microRNAs such as miR‐483‐5p and miR‐2861, which stimulate vascular calcification and bone marrow adipogenesis.^(^ ^54^ ^)^ These data support the role of senescence‐associated miRNAs and their transport by EVs as important pathways for age‐related bone loss. Of note, EVs from senescent BMSCs can also impact muscle satellite cells,^(^ ^55^ ^)^ underscoring the importance of bone‐muscle interactions. Suppressing the uptake of these BMSC‐derived EVs may have the potential to prevent sarcopenic changes in muscle.^(^ ^55^ ^)^

## Discussion and Conclusions

The field of geroscience has contributed a number of new and exciting discoveries to our understanding of musculoskeletal aging. Perhaps foremost among these is the recognition that cellular senescence increases with age in multiple tissues, including musculoskeletal tissues, contributing to organ dysfunction with aging. A key mechanism by which senescence impacts multiple organs and tissues is via the SASP, so that senescence is communicated to other cells and tissues in a paracrine manner. Although EVs are known to play an important role in cell–cell communication, their function as mediators of the SASP has only recently been acknowledged. This is due in large part to multiple studies indicating that senescent cells enhance their secretion of EVs relative to nonsenescent, healthy cells.^(^ ^15^, ^56^ ^)^ A broad examination of EV cargo catalogued in databases such as Vesiclepedia demonstrates that SASP factors, such as inflammatory cytokines and senescence‐associated miRNAs termed “GeroMirs”,^(^ ^9^ ^)^ are abundant in EVs. These data suggest that EVs are an essential component of the SASP and, thus, are likely to be important in age‐related musculoskeletal diseases. These SASP‐associated miRNAs detected in EVs may ultimately serve as useful biomarkers for frailty.^(^ ^57^ ^)^ Future research might be directed at better understanding the contribution(s) of various GeroMirs, in addition to miR‐34a, that are released from senescent muscle, bone, and cartilage cells via EVs.

The research reviewed here suggests that targeting senescent cells and their EV‐derived cargo may represent a potential therapeutic approach to treating age‐related musculoskeletal dysfunction. Indeed, such an approach for removing senescent cells (senolytic therapy) or modifying their secretory profile (senomorphic therapy) has already shown potential for preventing joint degeneration^(^ ^40^ ^)^ and age‐related bone loss.^(^ ^58^ ^)^ Other promising data suggest that additional small‐molecule therapies may support musculoskeletal health by targeting microRNA components of the SASP. As noted earlier, ovariectomy in rodents induces bone loss and increases circulating SASP‐associated miRNAs such as miR‐34a; however, the observed bone loss and increase in SASP‐associated miRNAs can be reduced with teriparatide or zolendronate treatment.^(^ ^53^ ^)^ Recent studies^(^ ^59^ ^)^ have shown that bone‐targeted EVs from BMSCs could play a role in osteoporosis therapy. When these BMSC‐derived EVs were combined with alendronate to generate Ale‐EVs, the targeting of EVs to bone via alendronate/hydroxyapatite binding was increased with low systemic toxicity.^(^ ^59^ ^)^ Another study also showed that EVs derived from genetically modified BMSCs (BMP2 overexpression) could be effective as biomimetic substitutes for growth factors to enhance bone regeneration in vivo.^(^ ^60^ ^)^ Another potential strategy is to modify the increase in EV secretion that occurs in senescent cells. A recent high‐throughput screen utilizing prostate cancer cells identified several existing small molecules that could be repurposed to either inhibit or stimulate exosome biogenesis.^(^ ^61^ ^)^ Future research might explore the repurposing of these drugs to prevent the release of EV‐derived, SASP‐associated miRNAs from senescent myotubes, chondrocytes, or osteocytes.

**Fig. 3:**
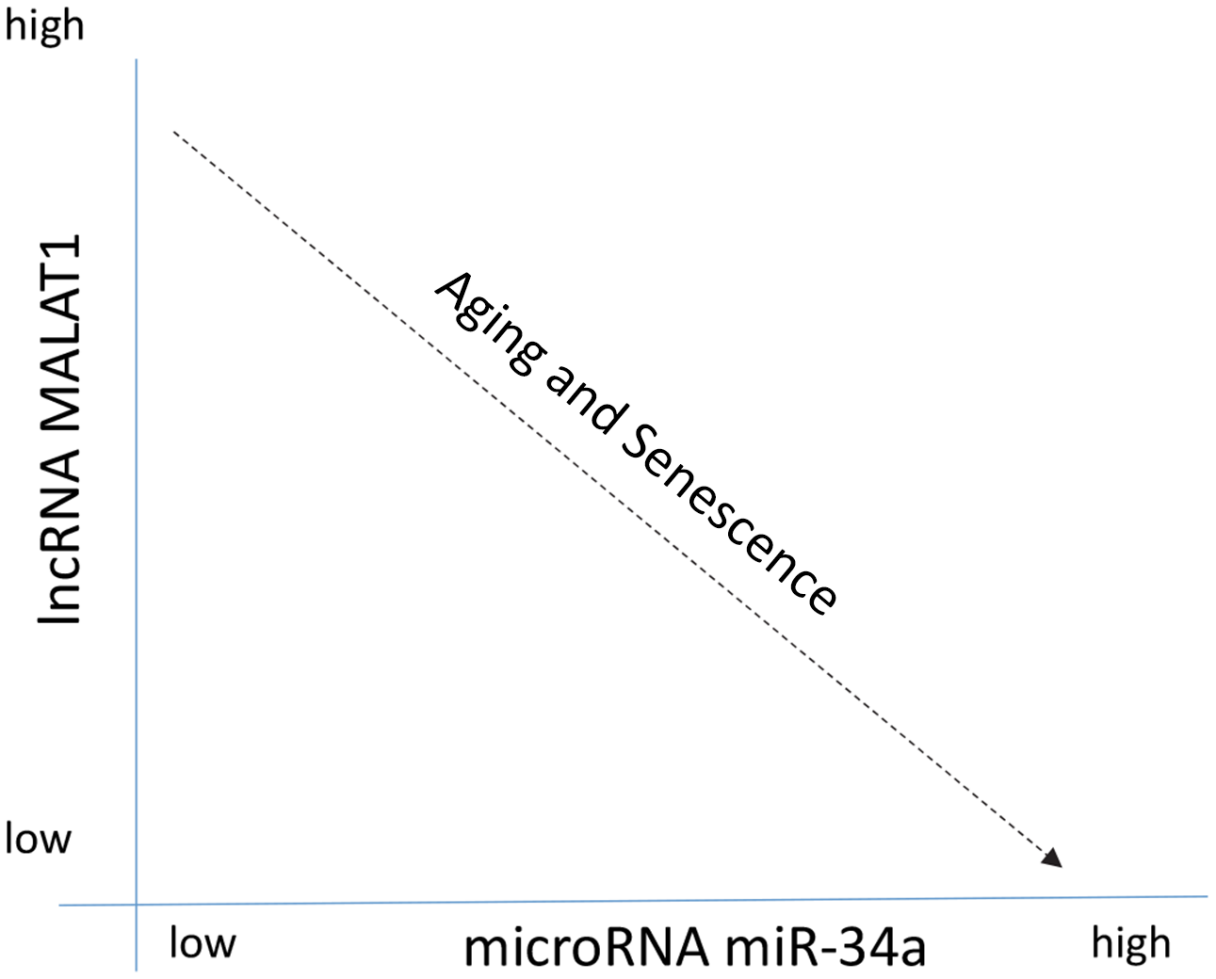
The senescence‐associated microRNA miR‐34a increases with age and senescence in multiple organs and tissues including skeletal muscle, cardiac muscle, and osteoarthritic cartilage. The competing endogenous long noncoding RNA MALAT1 can sponge miR‐34a, but MALAT1 decreases with age and with senescence. Interventions such as exercise in older adults can increase MALAT1 expression, potentially inhibiting miR‐34a and suppressing senescence and tissue dysfunction.

Another approach to preserve musculoskeletal function with age is to enhance the expression of endogenous antagonists to senescence‐associated, EV‐derived miRNAs. We have identified miR‐34a as one of these SASP‐related microRNAs in muscle, bone, and articular cartilage, whereas others have implicated miR‐34a in age‐related tissue dysfunction in the cardiovascular system^(^ ^28^, ^62^ ^)^ and brain.^(^ ^29^ ^)^ Long noncoding RNAs (lncRNAs) can function as “sponges” for microRNAs, and thus have a role as competing endogenous RNAs (ceRNAs). The long noncoding RNA MALAT1 is a sponge for miR‐34a,^(^ ^63^ ^)^ but MALAT1 is depleted in senescent cells.^(^ ^64^, ^65^ ^)^ MALAT1 itself appears to inhibit senescence, evident from experiments where MALAT1 siRNA increased senescent cell numbers in vitro.^(^ ^64^ ^)^ Importantly, MALAT1 decreases in patients with osteoarthritis,^(^ ^66^ ^)^ in patients with postmenopausal osteoporosis,^(^ ^67^ ^)^ and in mouse muscle atrophied with aging, hindlimb unloading, and glucocorticoid treatment^(^ ^63^, ^68^ ^)^ (Fig. 3). Exogenous application of MALAT1 in EVs can reduce inflammation and joint degeneration in an experimental model of OA.^(^ ^69^ ^)^ Together these findings suggest that interventions to increase MALAT1 in multiple tissues may have beneficial effects by sponging miR‐34a or suppressing its expression (Fig. 3). Of note, endurance and resistance exercise in older adults are associated with a significant increase in muscle MALAT1 expression.^(^ ^70^ ^)^ The studies reviewed here indicate that, although SASP factors carried by EVs are likely to play important roles in musculoskeletal diseases, several potential therapeutic approaches in development may be highly effective at inhibiting their activity to improve musculoskeletal health with aging.

## Author Contributions

MCA and SF prepared the sections on osteoarthritis, AAS contributed the section on bone, and MWH prepared the introduction, the section on muscle, and the discussion.

### Peer Review

The peer review history for this article is available at https://publons.com/publon/10.1002/jbm4.10686.

## References

[1] Briggs AM , Woolf AD , Dreinhöfer K , et al. Reducing the global burden of musculoskeletal conditions. Bull World Health Organ. 2018;96(5):366‐368. 29875522 10.2471/BLT.17.204891 PMC5985424

[2] Conboy IM , Rando TA . Heterochronic parabiosis for the study of the effects of aging on stem cells and their niches. Cell Cycle. 2012;11:2260‐2267. 22617385 10.4161/cc.20437 PMC3383588

[3] Rebo J, Mehdipour M, Gathwala R , et al. A single heterochronic blood exchange reveals rapid inhibition of multiple tissues by old blood. Nat Commun. 2016;7:13363. 27874859 10.1038/ncomms13363 PMC5121415

[4] Villeda SA, Plambeck K, Middeldorp J , et al. Young blood reverses age‐related impairments in cognitive function and synaptic plasticity in mice. Nat Med. 2014;20:659‐663. 24793238 10.1038/nm.3569 PMC4224436

[5] Saito Y , Yamagishi T , Nakamura T , et al. Klotho protein protects against endothelial dysfunction. Biochem Biophys Res Commun. 1998;248(2):324‐329. 9675134 10.1006/bbrc.1998.8943

[6] Yoshida M , Satoh A , Lin JB , et al. Extracellular vesicle‐contained eNAMPT delays aging and extends lifespan in mice. Cell Metab. 2019;30:329‐342.e5. 31204283 10.1016/j.cmet.2019.05.015 PMC6687560

[7] Sahu A , Clemens ZJ , Shinde SN , et al. Regulation of aged skeletal muscle regeneration by circulating extracellular vesicles. Nat Aging. 2021;1:1148‐1161. 35665306 10.1038/s43587-021-00143-2 PMC9165723

[8] Coppé JP , Desprez PY , Krtolica A , Campisi J . The senescence‐associated secretory phenotype: the dark side of tumor suppression. Annu Rev Pathol. 2010;5:99‐118. 20078217 10.1146/annurev-pathol-121808-102144 PMC4166495

[9] Caravia XM , López‐Otín C . Regulatory roles of miRNAs in aging. Adv Exp Med Biol. 2015;887:213‐230. 26662993 10.1007/978-3-319-22380-3_11

[10] Munk R , Panda AC , Grammatikakis I , Gorospe M , Abdelmohsen K . Senescence‐associated MicroRNAs. Int Rev Cell Mol Biol. 2017;334:177‐205. 28838538 10.1016/bs.ircmb.2017.03.008 PMC8436595

[11] Wang Z , Gao J , Xu C . Tackling cellular senescence by targeting miRNAs. Biogerontology. 2022;23(4):387‐400. 10.1007/s10522-022-09972-z. 35727469

[12] Kalra H , Simpson RJ , Ji H , Aikawa E , et al. Vesiclepedia: a compendium for extracellular vesicles with continuous community annotation. PLoS Biol. 2012;10(12):e1001450. 23271954 10.1371/journal.pbio.1001450 PMC3525526

[13] Kadota T , Fujita Y , Yoshioka Y , Araya J , Kuwano K , Ochiya T . Emerging role of extracellular vesicles as a senescence‐associated secretory phenotype: insights into the pathophysiology of lung diseases. Mol Asp Med. 2018;60:92‐103. 10.1016/j.mam.2017.11.005 29146100

[14] Jakhar R , Crasta K . Exosomes as emerging pro‐tumorigenic mediators of the senescence‐associated secretory phenotype. Int J Mol Sci. 2019;20(10):2547. 10.3390/ijms20102547 PMC6566274 31137607

[15] Basisty N , Kale A , Jeon OH , et al. A proteomic atlas of senescence‐associated secretomes for aging biomarker development. PLoS Biol. 2020;18(1):e3000599. 31945054 10.1371/journal.pbio.3000599 PMC6964821

[16] Fulzele S , Mendhe B , Khayrullin A , et al. Muscle‐derived miR‐34a increases with age in circulating extracellular vesicles and induces senescence of bone marrow stem cells. Aging. 2019;11(6):1791‐1803. 30910993 10.18632/aging.101874 PMC6461183

[17] Parker E , Mendhe B , Ruan L , et al. MicroRNA cargo of extracellular vesicles released by skeletal muscle fibro‐adipogenic progenitor cells is significantly altered with disuse atrophy. Physiol Genomics. 2022;54(8):296‐304. 35759450 10.1152/physiolgenomics.00177.2021 PMC9342138

[18] Jeon OH , Wilson DR , Clement CC , et al. Senescence cell‐associated extracellular vesicles serve as osteoarthritis disease and therapeutic markers. JCI Insight. 2019;4(7):e125019. 10.1172/jci.insight.125019 PMC6483636 30944259

[19] Forterre A , Jalabert A , Berger E , et al. Proteomic analysis of C2C12 myoblast and myotube exosome‐like vesicles: a new paradigm for myoblast‐myotube cross talk? PLoS One. 2014;9:e84153. 24392111 10.1371/journal.pone.0084153 PMC3879278

[20] Guescini M , Canonico B , Lucertini F , et al. Muscle releases alpha‐sarcoglycan positive extracellular vesicles carrying miRNAs in the bloodstream. PLoS One. 2015;10:e0125094. 25955720 10.1371/journal.pone.0125094 PMC4425492

[21] Whitham M , Parker BL , Friedrichsen M , et al. Extracellular vesicles provide a means for tissue crosstalk during exercise. Cell Metab. 2018;27:237‐251.e4. 29320704 10.1016/j.cmet.2017.12.001

[22] Bittel DC , Jaiswal JK . Contribution of extracellular vesicles in rebuilding injured muscles. Front Physiol. 2019;10:828. 31379590 10.3389/fphys.2019.00828 PMC6658195

[23] Podbielska M , Szulc ZM , Kurowska E , et al. Cytokine‐induced release of ceramide‐enriched exosomes as a mediator of cell death signaling in an oligodendroglioma cell line. J Lipid Res. 2016;57(11):2028‐2039. 27623848 10.1194/jlr.M070664 PMC5087870

[24] Trayssac M , Hannun YA , Obeid LM . Role of sphingolipids in senescence: implication in aging and age‐related diseases. J Clin Invest. 2018;128(7):2702‐2712. 30108193 10.1172/JCI97949 PMC6025964

[25] Trajkovic K , Hsu C , Chiantia S , et al. Ceramide triggers budding of exosome vesicles into multivesicular endosomes. Science. 2008;319(5867):1244‐1247. 18309083 10.1126/science.1153124

[26] Khayrullin A , Krishnan P , Martinez‐Nater L , et al. Very long‐chain C24:1 ceramide is increased in serum extracellular vesicles with aging and can induce senescence in bone‐derived mesenchymal stem cells. Cell. 2019;8(1):37. 10.3390/cells8010037 PMC6356348 30634626

[27] Zheng Y , Kong J , Li Q , Wang Y , Li J . Role of miRNAs in skeletal muscle aging. Clin Interv Aging. 2018;13:2407‐2419. 30538437 10.2147/CIA.S169202 PMC6254589

[28] Boon RA , Iekushi K , Lechner S , et al. MicroRNA‐34a regulates cardiac ageing and function. Nature. 2013;495:107‐110. 23426265 10.1038/nature11919

[29] Li QS , Cai D . Integrated miRNA‐Seq and mRNA‐Seq study to identify miRNAs associated with Alzheimer's disease using post‐mortem brain tissue samples. Front Neurosci. 2021;15:620899. 33833661 10.3389/fnins.2021.620899 PMC8021900

[30] Kukreti H , Amuthavalli K . MicroRNA‐34a causes ceramide accumulation and effects insulin signaling pathway by targeting ceramide kinase (CERK) in aging skeletal muscle. J Cell Biochem. 2020;121(5–6):3070‐3089. 32056304 10.1002/jcb.29312

[31] Parker E , Hamrick MW . Role of fibro‐adipogenic progenitor cells in muscle atrophy and musculoskeletal diseases. Curr Opin Pharmacol. 2021;58:1‐7. 33839480 10.1016/j.coph.2021.03.003 PMC8491779

[32] Sandonà M , Consalvi S , Tucciarone L , et al. HDAC inhibitors tune miRNAs in extracellular vesicles of dystrophic muscle‐resident mesenchymal cells. EMBO Rep. 2020;21(9):e50863. 32754983 10.15252/embr.202050863 PMC7507515

[33] Parker E , Khayrullin A , Kent A , et al. Hindlimb immobilization increases IL‐1β and Cdkn2a expression in skeletal muscle fibro‐Adipogenic progenitor cells: a link between senescence and muscle disuse atrophy. Front Cell Dev Biol. 2022;9:790437. 35047502 10.3389/fcell.2021.790437 PMC8762295

[34] Kang YJ , Yoo JI , Baek KW . Differential gene expression profile by RNA sequencing study of elderly osteoporotic hip fracture patients with sarcopenia. J Orthop Translat. 2021;29:10‐18. 34036042 10.1016/j.jot.2021.04.009 PMC8138673

[35] Withrow J , Murphy C , Liu Y , Hunter M , Fulzele S , Hamrick MW . Extracellular vesicles in the pathogenesis of rheumatoid arthritis and osteoarthritis. Arthritis Res Ther. 2016;18(1):286. 27906035 10.1186/s13075-016-1178-8 PMC5134070

[36] Murphy C , Withrow J , Hunter M , et al. Emerging role of extracellular vesicles in musculoskeletal diseases. Mol Asp Med. 2018;60:123‐128. 10.1016/j.mam.2017.09.006 PMC5856577 28965750

[37] Kolhe R , Hunter M , Liu S , et al. Gender‐specific differential expression of exosomal miRNA in synovial fluid of patients with osteoarthritis. Sci Rep. 2017;7(1):2029. 28515465 10.1038/s41598-017-01905-y PMC5435729

[38] Mihanfar A , Shakouri SK , Khadem‐Ansari MH , et al. Exosomal miRNAs in osteoarthritis. Mol Biol Rep. 2020;47(6):4737‐4748. 10.1007/s11033-020-05443-1. 32277444

[39] Endisha H , Datta P , Sharma A , et al. MicroRNA‐34a‐5p promotes joint destruction during osteoarthritis. Arthritis Rheumatol. 2021;73(3):426‐439. 33034147 10.1002/art.41552 PMC7986901

[40] Coryell PR , Diekman BO , Loeser RF . Mechanisms and therapeutic implications of cellular senescence in osteoarthritis. Nat Rev Rheumatol. 2021;17(1):47‐57. 33208917 10.1038/s41584-020-00533-7 PMC8035495

[41] Varela‐Eirín M , Carpintero‐Fernández P , Guitián‐Caamaño A , et al. Extracellular vesicles enriched in connexin 43 promote a senescent phenotype in bone and synovial cells contributing to osteoarthritis progression. Cell Death Dis. 2022;13:681. 35931686 10.1038/s41419-022-05089-w PMC9355945

[42] Qin W , Dallas SL . Exosomes and extracellular RNA in muscle and bone aging and crosstalk. Curr Osteoporos Rep. 2019;17(6):548‐559. 31741222 10.1007/s11914-019-00537-7 PMC7083338

[43] Robino JJ , Pamir N , Rosario S , et al. Spatial and biochemical interactions between bone marrow adipose tissue and hematopoietic stem and progenitor cells in rhesus macaques. Bone. 2020;133:115248. 31972314 10.1016/j.bone.2020.115248 PMC7085416

[44] Qin Y , Peng Y , Zhao W , et al. Myostatin inhibits osteoblastic differentiation by suppressing osteocyte‐derived exosomal microRNA‐218: a novel mechanism in muscle‐bone communication. J Biol Chem. 2017;292(26):11021‐11033. 28465350 10.1074/jbc.M116.770941 PMC5491785

[45] Davis C , Dukes A , Drewry M , et al. MicroRNA‐183‐5p increases with age in bone‐derived extracellular vesicles, suppresses bone marrow stromal (stem) cell proliferation, and induces stem cell senescence. Tissue Eng Part A. 2017;23(21–22):1231‐1240. 28363268 10.1089/ten.tea.2016.0525 PMC5689127

[46] Xu R , Shen X , Si Y , et al. MicroRNA‐31a‐5p from aging BMSCs links bone formation and resorption in the aged bone marrow microenvironment. Aging Cell. 2018;17(4):e12794. 29896785 10.1111/acel.12794 PMC6052401

[47] Li D , Liu J , Guo B , et al. Osteoclast‐derived exosomal miR‐214‐3p inhibits osteoblastic bone formation. Nat Commun. 2016;7:10872. 26947250 10.1038/ncomms10872 PMC4786676

[48] Fichtel P , von Bonin M , Kuhnert R , Möbus K , Bornhäuser M , Wobus M . Mesenchymal stromal cell‐derived extracellular vesicles modulate hematopoietic stem and progenitor cell viability and the expression of cell cycle regulators in an age‐dependent manner. Front Bioeng Biotechnol. 2022;10:892661. 35721867 10.3389/fbioe.2022.892661 PMC9198480

[49] Kulkarni R , Bajaj M , Ghode S , Jalnapurkar S , Limaye L , Kale VP . Intercellular transfer of microvesicles from young mesenchymal stromal cells rejuvenates aged murine hematopoietic stem cells. Stem Cells. 2018;36(3):420‐433. 29230885 10.1002/stem.2756

[50] Zhang F , Cui J , Liu X , et al. Roles of microRNA‐34a targeting SIRT1 in mesenchymal stem cells. Stem Cell Res Ther. 2015;6:195. 26446137 10.1186/s13287-015-0187-x PMC4597437

[51] Chen L , Holmstrøm K , Qiu W , et al. MicroRNA‐34a inhibits osteoblast differentiation and in vivo bone formation of human stromal stem cells. Stem Cells. 2014;32(4):902‐912. 24307639 10.1002/stem.1615

[52] Nóbrega OT , Morais‐Junior GS , Viana NI , et al. Circulating miR‐34a and bone mineral density of Brazilian very‐old adults. J Aging Res. 2020;(2020):3431828. 32377434 10.1155/2020/3431828 PMC7196151

[53] Weigl M , Kocijan R , Ferguson J , et al. Longitudinal changes of circulating miRNAs during bisphosphonate and Teriparatide treatment in an animal model of postmenopausal osteoporosis. J Bone Miner Res. 2021;36(6):1131‐1144. 33598975 10.1002/jbmr.4276 PMC8252367

[54] Wang ZX , Luo ZW , Li FX , et al. Aged bone matrix‐derived extracellular vesicles as a messenger for calcification paradox. Nat Commun. 2022;13(1):1453. 35304471 10.1038/s41467-022-29191-x PMC8933454

[55] Dai H , Zheng W , Luo J , et al. Inhibiting uptake of extracellular vesicles derived from senescent bone marrow mesenchymal stem cells by muscle satellite cells attenuates sarcopenia. J Orthop Translat. 2022;35:23‐36. 35846725 10.1016/j.jot.2022.06.002 PMC9260455

[56] Boulestreau J , Maumus M , Rozier P , Jorgensen C , Noël D . Mesenchymal stem cell derived extracellular vesicles in aging. Front Cell Dev Biol. 2020;8:107. 32154253 10.3389/fcell.2020.00107 PMC7047768

[57] Rusanova I , Fernández‐Martínez J , Fernández‐Ortiz M , et al. Involvement of plasma miRNAs, muscle miRNAs and mitochondrial miRNAs in the pathophysiology of frailty. Exp Gerontol. 2019;124:110637. 31199979 10.1016/j.exger.2019.110637

[58] Farr JN , Xu M , Weivoda MM , et al. Targeting cellular senescence prevents age‐related bone loss in mice. Nat Med. 2017;23(9):1072‐1079. 28825716 10.1038/nm.4385 PMC5657592

[59] Wang Y , Yao J , Cai L , et al. Bone‐targeted extracellular vesicles from mesenchymal stem cells for osteoporosis therapy. Int J Nanomedicine. 2020;15:7967‐7977. 33116512 10.2147/IJN.S263756 PMC7573321

[60] Huang CC , Kang M , Lu Y , et al. Functionally engineered extracellular vesicles improve bone regeneration. Acta Biomater. 2020;109:182‐194. 32305445 10.1016/j.actbio.2020.04.017 PMC8040700

[61] Datta A , Kim H , McGee L , et al. High‐throughput screening identified selective inhibitors of exosome biogenesis and secretion: a drug repurposing strategy for advanced cancer. Sci Rep. 2018;8(1):8161. 29802284 10.1038/s41598-018-26411-7 PMC5970137

[62] Raucci A , Macrì F , Castiglione S , Badi I , Vinci MC , Zuccolo E . MicroRNA‐34a: the bad guy in age‐related vascular diseases. Cell Mol Life Sci. 2021;78(23):7355‐7378. 34698884 10.1007/s00018-021-03979-4 PMC8629897

[63] Ruan L , Mendhe B , Parker E , et al. Long non‐coding RNA MALAT1 is depleted with age in skeletal muscle in vivo and MALAT1 silencing increases expression of TGF‐β1 in vitro. Front Physiol. 2022;12:742004. 35126169 10.3389/fphys.2021.742004 PMC8814451

[64] Abdelmohsen K , Panda A , Kang MJ , et al. Senescence‐associated lncRNAs: senescence‐associated long noncoding RNAs. Aging Cell. 2013;12(5):890‐900. 23758631 10.1111/acel.12115 PMC3773026

[65] Tripathi V , Shen Z , Chakraborty A , et al. Long non‐coding RNA MALAT1 controls cell cycle progression by regulating the expression of oncogenic transcription factor B‐MYB. PLoS Genet. 2013;9:e1003368. 23555285 10.1371/journal.pgen.1003368 PMC3605280

[66] Giordano R , Petersen KK , Santoro M , et al. Circulating long non‐coding RNA signature in knee osteoarthritis patients with postoperative pain one‐year after total knee replacement. Scand J Pain. 2021;21(4):823‐830. 34323060 10.1515/sjpain-2021-0069

[67] Qian TY , Wan H , Huang CY , Hu XJ , Yao WF . Plasma LncRNA MALAT1 expressions are negatively associated with disease severity of postmenopausal osteoporosis. Lab Med. 2022;53(5):446‐452. 35311990 10.1093/labmed/lmac009

[68] Hitachi K , Nakatani M , Funasaki S , et al. Expression levels of long non‐coding RNAs change in models of altered muscle activity and muscle mass. Int J Mol Sci. 2020;21:1628. 10.3390/ijms21051628 PMC7084395 32120896

[69] Pan C , Huang W , Chen Q , et al. LncRNA Malat‐1 from MSCs‐derived extracellular vesicles suppresses inflammation and cartilage degradation in osteoarthritis. Front Bioeng Biotechnol. 2021;9:772002. 34976968 10.3389/fbioe.2021.772002 PMC8715093

[70] De Sanctis P , Filardo G , Abruzzo PM , et al. Non‐coding RNAs in the transcriptional network that differentiates skeletal muscles of sedentary from long‐term endurance‐ and resistance‐trained elderly. Int J Mol Sci. 2021;22:1539. 33546468 10.3390/ijms22041539 PMC7913629

